# Chemical-Mediated Microbial Interactions Can Reduce the Effectiveness of Time-Series-Based Inference of Ecological Interaction Networks

**DOI:** 10.3390/ijerph19031228

**Published:** 2022-01-22

**Authors:** Kenta Suzuki, Masato S. Abe, Daiki Kumakura, Shinji Nakaoka, Fuki Fujiwara, Hirokuni Miyamoto, Teruno Nakaguma, Mashiro Okada, Kengo Sakurai, Shohei Shimizu, Hiroyoshi Iwata, Hiroshi Masuya, Naoto Nihei, Yasunori Ichihashi

**Affiliations:** 1 BioResource Research Center, RIKEN, Tsukuba 305-0074, Japan; hiroshi.masuya@riken.jp (H.M.); yasunori.ichihashi@riken.jp (Y.I.); 2 Center for Advanced Intelligence Project, RIKEN, Chuo-ku, Tokyo 103-0027, Japan; masato.abe@riken.jp (M.S.A.); shohei-shimizu@biwako.shiga-u.ac.jp (S.S.); 3 Graduate School of Life Science, Hokkaido University, Sapporo 060-0810, Japan; dkumakura@eis.hokudai.ac.jp (D.K.); snakaoka@sci.hokudai.ac.jp (S.N.); 4 Laboratory of Mathematical Biology, Faculty of Advanced Life Science, Hokkaido University, Sapporo 060-0819, Japan; 5 Graduate School of Agricultural and Life Sciences, The University of Tokyo, Bunkyo-ku, Tokyo 113–8657, Japan; fukifujiwara9113@g.ecc.u-tokyo.ac.jp (F.F.); mashiro@ut-biomet.org (M.O.); sakurai@ut-biomet.org (K.S.); iwata@ut-biomet.org (H.I.); 6 Graduate School of Horticulture, Chiba University, Matsudo 271-8501, Japan; h-miyamoto@faculty.chiba-u.jp (H.M.); h-nakaguma@sermas.co.jp (T.N.); 7 RIKEN Center for Integrative Medical Sciences, Yokohama 230-0045, Japan; 8 Sermas Co., Ltd., Ichikawa 272-0015, Japan; 9 Faculty of Food and Agricultural Sciences, Fukushima University, Fukushima 960-1296, Japan; nihei@agri.fukushima-u.ac.jp

**Keywords:** chemical-mediated interactions, ecological interaction network, microbiome, exometabolome, mediator-explicit model, interaction network inference, microbial time series

## Abstract

Network-based assessments are important for disentangling complex microbial and microbial–host interactions and can provide the basis for microbial engineering. There is a growing recognition that chemical-mediated interactions are important for the coexistence of microbial species. However, so far, the methods used to infer microbial interactions have been validated with models assuming direct species-species interactions, such as generalized Lotka–Volterra models. Therefore, it is unclear how effective existing approaches are in detecting chemical-mediated interactions. In this paper, we used time series of simulated microbial dynamics to benchmark five major/state-of-the-art methods. We found that only two methods (CCM and LIMITS) were capable of detecting interactions. While LIMITS performed better than CCM, it was less robust to the presence of chemical-mediated interactions, and the presence of trophic competition was essential for the interactions to be detectable. We show that the existence of chemical-mediated interactions among microbial species poses a new challenge to overcome for the development of a network-based understanding of microbiomes and their interactions with hosts and the environment.

## 1. Introduction

There is a growing recognition that microbiome science needs to move beyond descriptive studies to a more systematic understanding that would facilitate mechanical, predictive, and manipulative approaches to rational microbial engineering [1]. Network-based approaches will help disentangle complex microbial and microbe-host/environment interactions, which could have applications universally applicable to medicine and health care, agriculture, and other environmental and industrial areas [2,3,4]. Current attempts to understand interaction networks combine comprehensive quantification of microbiota using next-generation sequencing technologies with various network inference methodologies based on statistical and machine learning approaches.

Recent studies revealed that the exchange of metabolites plays an essential role in microbial interactions. All microorganisms exchange metabolites, such as vitamins, amino acids, nucleotides, or growth factors, by releasing them into the surrounding environment [5,6,7]. This metabolite cross-feeding is common both among different bacterial species and between bacteria and members of other kingdoms [6]. In consequence, a microbial community forms a unique chemical environment known as the exometabolome, which comprises hundreds of metabolites, the majority of which are derived from living cells [7,8]. Considering its prominent role in microbial interactions, the exometabolome and chemical-mediated interactions would be tightly linked with the dynamics of microbial communities, including composition, stability, and functionality.

However, most of the benchmarking of methods proposed for inferring microbial interactions has been performed using generalized Lotka–Volterra (gLV) models, which assumes direct (species-species) interactions [9,10,11]. Some methods were developed based on the gLV equation itself [12,13,14,15]. Recent studies [16,17,18] revealed that the species-species interaction models are insufficient to capture dynamics that occur through chemical-mediated interactions, while such interactions would serve a prominent role in the coexistence of diverse microbial species [19]. Therefore, benchmarking with the direct interaction model alone would be insufficient. If the presence of chemical-mediated interactions reduces the reliability of the network inference methods, it poses a new challenge for studies of microbial interaction networks.

In this paper, we investigated how accurate existing time-series-based inferences of ecological interactions would be when underlying interactions are mediated by chemicals. For this purpose, we started with an in-silico mediator-explicit model of microbial population dynamics whose parameter values had been experimentally calibrated [19]. We compared the performance of five major/state-of-the-art methods under different model assumptions, including the process of direct or chemical-mediated interactions, the presence or absence of competition for nutrients, as well as the effect of different sampling intervals and magnitudes of stochasticity.

## 2. Materials and Methods

### 2.1. Models

Our model is based on the mediator-explicit model proposed and validated for actual microbial systems by Niehaus et al. [19]. In this model, each species can produce multiple chemicals, and each chemical can influence multiple species. Each influence is represented by a coefficient defined for pairs of species and chemicals. The mediator-explicit model (*M*) is defined as follows:

(1) $$dx_{i}/dt=F_{M}\left(x_{i}\right)=r_{i}\left(1-x_{i}/K\right)x_{i}+\Sigma_{k}\left(\rho_{ik}{}^{+}\frac{c_{k}}{c_{k}+\kappa_{ik}}-\rho_{ik}{}^{-}\frac{c_{k}}{c_{k}+\kappa_{ik}}\right)x_{i}-\delta x_{i},$$

(2) $$dc_{k}/dt=G_{M}\left(c_{k}\right)=\Sigma_{i}\left(\beta_{ki}x_{i}-\alpha_{ki}x_{i}\frac{c_{k}}{c_{k}+\kappa_{ik}}\right)-\delta c_{k}.$$

Here, variables $x_{i}$ and $c_{k}$ are the abundance of microbes and the amount of chemicals, respectively, and i=1,2,…,n and k=1,2,…,m, i.e., we assume a system consisting of n microbes and m chemicals. $r_{i}$ is the intrinsic growth rate, K is the carrying capacity, $\kappa_{ik}$ is the half-saturation density of the effect of chemical k on species i, $\rho_{ik}{}^{+}$ and $\rho_{ik}{}^{-}$ represent the positive and negative effect of chemical k on species i, respectively, $\alpha_{ki}$ is the maximum rate of consumption of chemical k by species i, $\beta_{ki}$ is the rate of production of chemical k by species i, and δ is the constant dilution rate (see Table 1 for the parameter values). We also use the matrix representation of parameters as follows: n×m matrices $\kappa =\left\{\kappa_{ik}\right\}$, $\rho^{+}=\left\{\rho_{ik}{}^{+}\right\}$ and $\rho^{-}=\left\{\rho_{ik}{}^{-}\right\}$, and m×n matrices $\alpha =\left\{\alpha_{ki}\right\}$ and $\beta =\{\beta_{ki}\}$. While Niehaus et al. [19] did not include the carrying capacity, we introduced it to avoid overflow in numerical calculations. Moreover, it is reasonable to assume that population growth is limited due to resource availability. Model M assumes that each species has an independent bound for their population growth, i.e., there is no competition for resources among species.

In addition to model *M*, we introduce model *M*′ that includes the effect of competition for nutrients:

(3) $$dx_{i}/dt=F_{M^{\prime }}\left(x_{i}\right)=r_{i}\left(1-\Sigma_{j}x_{j}/nK\right)x_{i}+\Sigma_{k}\left(\rho_{ik}+\frac{c_{k}}{c_{k}+\kappa_{ik}}-\rho_{ik}-\frac{c_{k}}{c_{k}+\kappa_{ik}}\right)x_{i}-\delta x_{i},$$

(4) $$dc_{k}/dt=G_{M^{\prime }}\left(c_{k}\right)=G_{M}\left(c_{k}\right).$$

This model assumes that all species equally depend on all available nutrients, and therefore the increase of any species reduces the growth rate of other species. To be consistent with model M, we assumed that the total abundance $\Sigma_{j}x_{j}$ is bounded by nK.

As the third and fourth models, we considered models with direct interactions comparable to the models *M* and *M*′. These models are introduced based on the following considerations. Since the overall effect of chemicals on the species’ growth rate is $\rho =\rho^{+}-\rho^{-}$ and the rate of production of chemicals by each species is β, ρβ represents the instantaneous effects of a species on another, which is comparable to the direct interactions represented by an interaction matrix $A=\left\{a_{ij}\right\}$. Therefore, we defined model *D* as

(5) $$dx_{i}/dt=F_{D}\left(x_{i}\right)=r_{i}\left(1-x_{i}/K\right)x_{i}+\Sigma_{j}a_{ij}x_{i\;}x_{j}-\delta x_{i},$$

and the model *D*′ as

(6) $$dx_{i}/dt=F_{D^{\prime }}\left(x_{i}\right)=r_{i}\left(1-\Sigma_{j\;}x_{j}/nK\right)x_{i}+\Sigma_{j}a_{ij}x_{i\;}x_{j}-\delta x_{i}.$$

These models are simpler than models *M* and *M*′ in that they do not include the effects of the consumption of chemicals by microbes.

To obtain the time-series data, we introduced stochasticity and small influx to the above models and obtained numerical solutions using the Euler–Maruyama Scheme:

(7) $$x_{i}\left(t+1\right)=x_{i}\left(t\right)+F_{\Omega }△t+\sigma \sqrt{△t}△W_{t}+\epsilon .$$

Here, △t is the step size of numerical simulation and is fixed as △t=0.025, $△W_{t}$ is a normal distribution with s.d. of 1, σ represents the magnitude of noise, and Ω is $\Omega \in \left\{M,M^{\prime },D,D^{\prime }\right\}$. During the numerical simulations, some species would approach zero infinitely. Thus, to avoid underflow, we introduced a small influx ϵ. The parameter values are scaled so that △t=1 corresponds to 1 day and K=1 corresponds to $10^{7}$ individuals/mL.

### 2.2. Effective Interaction Matrix

In models *M* and *M*′, it is necessary to interpret the microbial interactions that occur via chemicals as direct interactions to evaluate the network inference methods. For this purpose, we first combine Equations (1) and (2) and describe them as $\left(dx_{i}/dt,\ldots ,dc_{k}/dt,\ldots \right)=\left(F_{M}\left(x_{i}\right),\ldots ,G_{M}\left(c_{k}\right)\right)$. Under this expression, the Jacobi matrix can be written as

(8) $$J_{M}=\left(\begin{array}{cccc} \partial F_{M}\left(x_{1}\right)/\partial x_{1} & \cdots & \partial F_{M}\left(x_{1}\right)/\partial c_{1} & \cdots \\ \vdots & \ddots & \vdots & \ddots \\ \partial G_{M}\left(c_{1}\right)/\partial x_{1} & \cdots & \partial G_{M}\left(c_{1}\right)/\partial c_{1} & \cdots \\ \vdots & \ddots & \vdots & \ddots \end{array}\right)=\left(\begin{array}{cc} J_{F_{M}}{}^{\left(x\right)} & J_{F_{M}}{}^{\left(c\right)}\\ J_{G_{M}}{}^{\left(x\right)} & J_{G_{M}}{}^{\left(c\right)} \end{array}\right)$$

Here, the m×n block, $J_{F_{M}}{}^{\left(c\right)}$, contains the positive and negative effects of chemicals on organisms, and the n×m block, $J_{G_{M}}{}^{\left(x\right)}$, contains the effects of production and consumption by microbes on chemicals. Then, we interpret $A^{*}=J_{F_{M}}{}^{\left(c\right)}J_{G_{M}}{}^{\left(x\right)}$ as a matrix representing the effective microbial interactions (effective interaction matrix), which is the target of the evaluation of the network inference methods. The above argument holds for *M*′ as well. In models *D* and *D*′, the non-zero part of the Jacobi matrix is consistent with that of A. Therefore, in *D* and *D*′, we identify A with $A^{*}$.

### 2.3. Data Preparation

**Figure 1:**
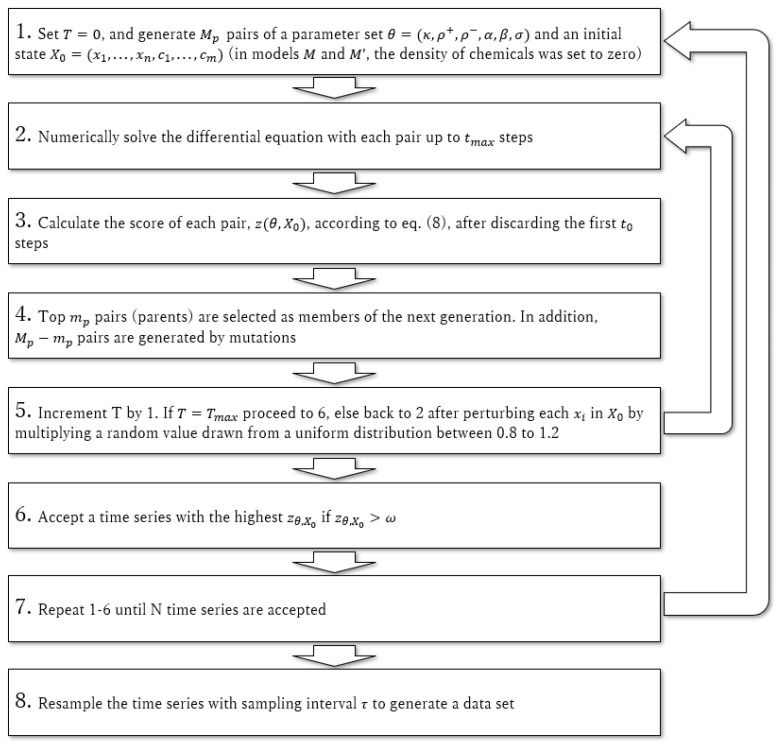
Procedure for generating a data set.

For each model, we generated the time series for generating data sets using pairs of a parameter set $\theta =\left(\kappa ,\rho^{+},\rho^{-},\alpha ,\beta ,\sigma \right)$ and an initial state $X_{0}=\left(x_{1},\ldots ,x_{n},c_{1},\ldots ,c_{m}\right)$. Since it was difficult to obtain multispecies coexistence with the initial parameter set, these pairs are obtained by several optimization steps as follows (see Figure 1 for a summary of the procedure and Table 2 for the parameter values). At T=0, we initialized $M_{p\;}$ pairs of a parameter set and an initial state. For the initial states, abundances of microbes were drawn from a uniform distribution between 10ϵ and 100ϵ, and in models M and $M^{\prime }$, the density of chemicals was set to zero. Then, we numerically solved the differential equation with each pair up to $t_{max}$ steps (corresponding to time series of $t_{max}\Delta t$ days). After discarding the first $t_{0}$ steps, we calculated the score of each pair according to the following evaluation function:

(9) $$z_{\theta ,X_{0}}=S^{*}D\left(4c-4c^{2}\right).$$

Here, denoting the time-averaged abundance of species i in the time series as ${\hat{x}}_{i}$, $S^{*}$ is the number of “major” species (number of species whose ${\hat{x}}_{i}$ is larger than η), $D=\overset{n}{\underset{i=1}{\sum }}{\left({\hat{x}}_{i}/\overset{n}{\underset{j=1}{\sum }}{\hat{x}}_{j}\right)}^{1/2}$ is the Simpson’s diversity index, and c=L/n(n−1) is the connectance (L is the number of non-zero values for off-diagonal elements in an interaction matrix). D and c were calculated for the major species. This function takes its maximum value when the number of major species is n, their time-averaged abundance is equal, and the connectance is 0.5. We adopted the evaluation function to obtain a state where the number of coexisting species is as high as possible, their interactions are neither too sparse nor too dense, and a small number of species are not dominant. Then, top $m_{p}$ pairs (parents) are selected as members of the next generation in addition to $M_{p}-m_{p}$ pairs that are generated by mutations, where one of the non-zero elements of a matrix in θ is selected and switched with another element of the same matrix; this procedure is repeated one to four times with equal probability. Then, $M_{p}$ pairs are again evaluated as above. However, at each numerical simulation, each $x_{i}$ in $X_{0}$ are perturbed by multiplying a random value drawn from a uniform distribution between 0.8 to 1.2. After repeating the same procedure for $T_{max}$ steps, a pair with the highest $z_{\theta ,X_{0}}$ is accepted if $z_{\theta ,X_{0}}>\omega$. This procedure is repeated until we obtain N accepted time series for each model.

We obtained the data sets for testing the network inference methods by generating time series with various noise magnitude σ and resampling them at different intervals τ. To evaluate the effect of the sampling interval, we first generated four data sets from a time series of a fixed noise magnitude σ=1 and sampled 100 of the last points with different intervals τ∈{10,20,40,80} (this corresponds to time series of 25, 50, 100 and 200 days sampled every 0.25, 0.5, 1 and 2 days). Then, to evaluate the effect of noise magnitude, we generated 4 data sets from time series of different noise magnitude σ∈{0.5,1,2,4} and sampled 100 of the last points with a fixed interval τ=40. Basic characteristics of the communities obtained under different noise magnitude are described in Appendix B.

### 2.4. Network Inference Methods

We compared five methods (Pearson and Spearman rank correlation, LSA, CCM, and LIMITS) in this paper. Taking multivariate time series as input values, these methods return matrices of statistics indicating the presence/absence of interaction between species pairs, represented by correlation coefficients in Pearson, Spearman, and LSA, predictive skills in CCM, and interaction coefficients in LIMITS. These matrices are accompanied by matrices of *p*-values, and these can also be used to detect interactions. Except for the statistics of LIMITS, the matrices are dense such that every element has a non-zero value.

We refer to such a matrix as the inferred interaction matrix and consider the statistic and *p*-value of each method as a classifier to predict the presence or absence of effect from one species to another. Interactions are represented by non-zero values of the non-diagonal elements of an actual/inferred interaction matrix. When considering the presence or absence of an interaction, it is necessary to distinguish between the direction of the interaction. In the following, in addition to the term “interaction”, we regard the presence or absence of effect from species A to B and that of species B to A if strict consideration of directionality is needed.

#### 2.4.1. Pearson and Spearman Correlation Coefficient

Pearson correlation coefficient [20] is a measure of linear correlation between two variables. It is a parametric measure that assumes Gaussian distributions of variables. Spearman rank correlation coefficient [21] is a nonparametric measure of rank correlation. It is relevant even if both or one of the variables is non-Gaussian and thus is more broadly applicable than Pearson correlation. These correlation coefficients are frequently used for the network-based analysis of biological systems [9]. Both methods provide correlation coefficients between −1 and 1 for each pair of variables, accompanied by the *p*-values. The correlation matrix and *p*-values are symmetric, i.e., they are inherently imprecise if the actual ecological interactions involve asymmetric interactions.

#### 2.4.2. Local Similarity Analysis (LSA)

LSA (see Ruan et al. [22] for detail; also refer to Beman et al., [23], Steele et al. [24], Xia et al. [25]) also considers the association between time series but is optimized to detect non-linear, time-sensitive relationships. It captures local and potentially time-delayed association patterns that cannot be identified by ordinary correlation analysis [25]. A previous study [9] showed that, for Lotka–Volterra sparse ecological relationships, it attains greater performance than other correlation-based approaches. We performed LSA analysis according to the implementation by the authors [22] and calculated the *p*-value with 2000 bootstrap samples. This method returns a correlation coefficient between −1 and 1 for each pair of variables, accompanied by the *p*-values. The correlation matrix and *p*-values are symmetric, i.e., it has the same problem as Pearson and Spearman rank correlation.

#### 2.4.3. Convergent Cross Mapping (CCM)

CCM [26] is a statistical test for a causal relationship between two time series variables and attempts to address the problem that correlation is often not an indicator of the presence or absence of actual causal relationships. This method is based on Takens’ embedding theorem [27], which states that the essential information of a multi-dimensional dynamical system is retained in the time series of any single variable of that system. Based on this theory, in a pair of time series (X, Y), if X has a high forecasting skill in predicting Y, causality will be detected in the direction of Y → X. The predictive skill, denoted as $\rho_{Y\to X}$, is quantified by the Pearson correlation coefficient between actual Y and Y predicted by X. Different from the previous correlation-based methods, $\rho_{Y\to X}$ is usually unequal to $\rho_{X\to Y}$. Its *p*-value is calculated by the bootstrapping procedure to account for the effects of the predictability inherent in the target time series (such as periodicity). To apply this method to systematic network inference, we needed to extend the implementation by the authors (Appendix A).

#### 2.4.4. LIMITS

LIMITS [12] is based on the generalized Lotka–Volterra difference equation. The algorithm combines forward stepwise regression and bootstrap aggregation (bagging) to determine, in a majority voting fashion, the pairs of interacting variables and the value of their interaction coefficient. For each species, the logarithm of the change in abundance per time step is used as the response variable. Then, this method selects variables to be explanatory variables of linear regression step by step, as long as the predictive performance is improved. The species selected as the explanatory variables are candidates for the interaction partners. This process is repeated (here, 500 times) as bootstrap replicates, and the species selected in more than half of all iterations are finally determined as the interaction partners, i.e., variables that can affect the response variable. The interaction is estimated asymmetrically and in a way that includes positive and negative values. For each pair of species, the *p*-value was obtained as the number of bootstrap trials in which the interaction coefficient was zero divided by the number of all trials. This method returns a sparse matrix for the interaction matrix, but that of the *p*-values can be dense.

### 2.5. Evaluation

Since the number of major species varies from trial to trial, we evaluated only the interaction network of the top five abundant species. Due to the condition for acceptance (ω=5 in $z\left(\theta ,X_{0}\right)>\omega$), every trial contains at least 5 major species (Appendix B).

We here focus on the performance of the above methods in detecting the interactions because, in both real ecological communities and our model, interactions between species are sparse (Appendix B). To evaluate the performance of network inference methods through a classifier (correlation coefficient, predictive skill, or interaction coefficient), we used ROC-AUC (the area under the curve of a receiver-operator characteristic curve; Supporting Figure S1). An ROC curve is the plot of (1-*specificity*, *sensitivity*). Here, *sensitivity* is the number of interacting species pairs that a method was able to find divided by the actual number of them, and *specificity* is the number of non-interacting species pairs that a method correctly omitted divided by the actual number (in both cases, the presence/absence of an effect from species A to B and B to A must be distinguished). Given a matrix of statistics, an ROC curve is obtained when we keep changing the threshold value by which we judge that an interaction exists between species pairs. When the threshold value is too high, none of the elements are accepted, and the value takes (0,0). When the threshold value is too low, all of the elements are accepted, and the value takes (1,1). (This explanation is valid for cases where larger values indicate a greater likelihood that an interaction exists, but the opposite is true for cases such as *p*-values where smaller values suggest the presence of an interaction.) When the threshold is varied over a range covering (0,0) to (1,1), the more convex the ROC curve is to the upper left (in other words, the more sensitivity can be increased without decreasing specificity), the better the statistic is at detecting interactions. Here, the value of the ROC’s AUC varies from 0 to 1 and becomes 1 when a method performs the best and 0.5 when it is indifferent to random selection.

The ROC-AUC can evaluate the overall performance even when the optimal threshold is not known, but from a practical standpoint, it is useful to have evaluations obtained under certain conditions. For this reason, we evaluated the *precision* for the interactions when half of the matrix elements are selected according to the descending (ascending) order (for simplicity, we refer to it as Precision (*c* = 0.5)), that is, how many correct interaction pairs are contained in the top 50% of candidates.

### 2.6. Software

We used Mathematica 10.2 and 11.0 for our analysis. The Mathematica notebook files used for the analysis can be downloaded from: https://drive.google.com/file/d/11suHcj6RCC2p6gQ6zjTvzbo-DeExzR-g/view?usp=sharing (accessed on 19 January 2022).

## 3. Results

**Figure 2:**
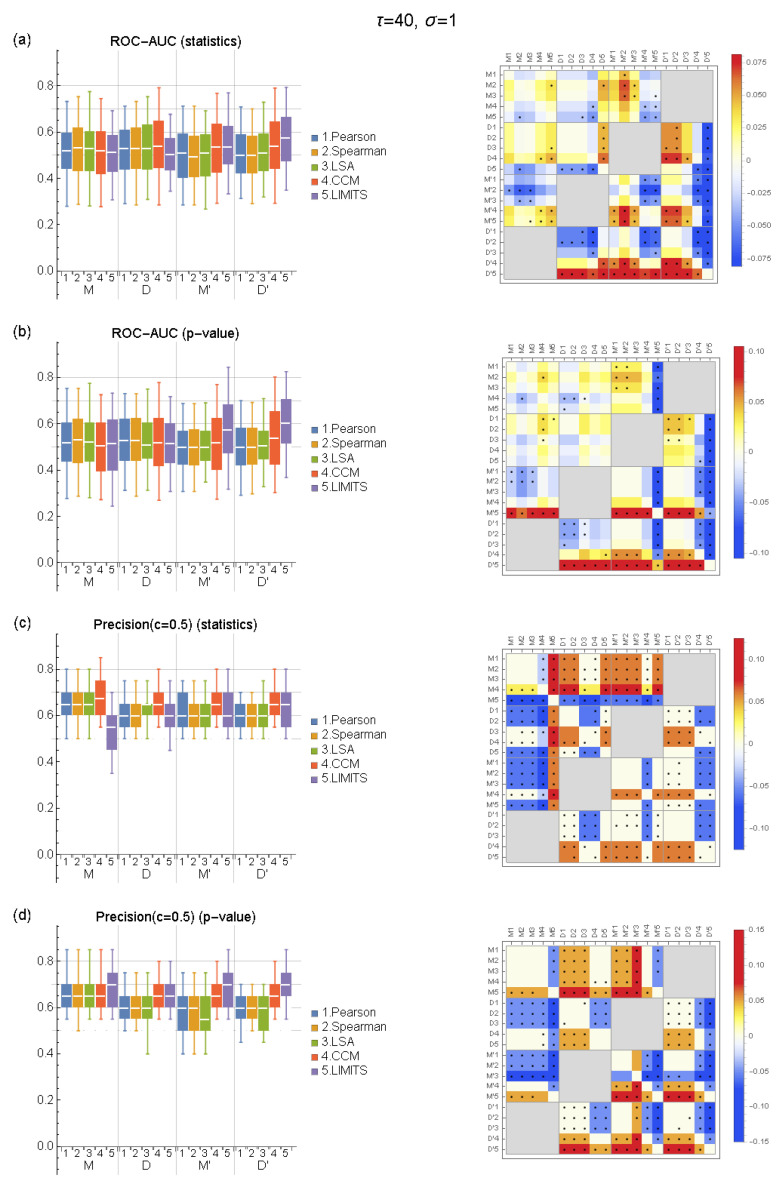
Performance of network inference methods for different models (**left**) and the comparison of the different pairs of model and method (**right**) for τ=1 and σ=1. (**a**,**b**) ROC-AUC of networks inferred by the statistics and *p*-values of each method, respectively, and (**c**,**d**) precision (c=0.5) of networks inferred by the statistics and *p*-values of each method, respectively. In the box plot, white lines indicate the median, box edges indicate the first and third quartile value, and whiskers indicate maximum and minimum values. The heatmap on the right side of each panel aids in comparison between the different pairs of models/methods. The value of a cell is obtained by subtracting the median of the pair of models/method in a column from the same value of the pair in a row. Black dots indicate that the difference is significant (p<0.05) based on Mann–Whitney test. We compared the performance of the different methods applied to the same model, and we compared the performance when the condition of competition was the same but the property of the interaction was different (*M* and *D* or *M*′ and *D*′) and when the property of the interaction was the same but the condition of competition was different (*M* and *M*′ or *D* and *D*′).

**Figure A1:**
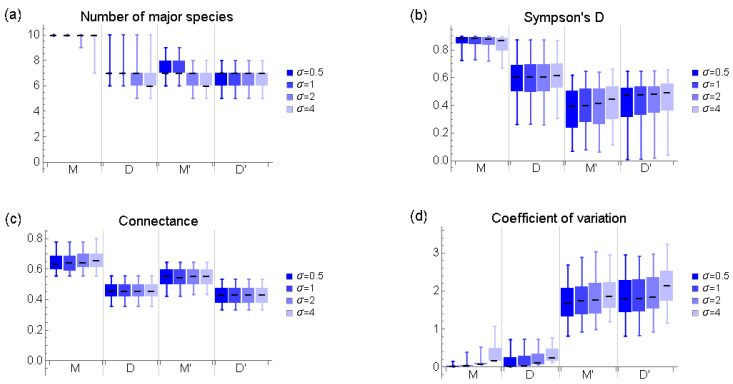
Basic characteristics of the communities we used for the evaluation. The black line indicates median, the box edges indicate first and third quartile values, and whiskers indicate maximum and minimum values. (**a**) The number of major species, (**b**) Simpson’s diversity index, (**c**) connectance of effective interaction matrix, and (**d**) coefficient of variation.

To evaluate five network inference methods, we generated in silico time-series microbial population datasets based on the mediator-explicit model (*M*) and direct interaction model (*D*) as well as modified models by adding the effect of resource competition (*M*′ and *D*′) (Appendix B; Figure A1). For the four models *M*, *D*, *M*′, and *D*′, we obtained network estimation results with five methods under different simulation conditions (see Supporting Figures S2–S5 for representative results). Results from the ROC-AUC indicate that in the presence of resource competition (*M*′ and *D*′), LIMITS and CCM, in this order, performed better than other methods (Figure 2a,b). While LIMITS attained the highest performance when *p*-values were used to detect interactions (Figure 2b), when the interaction was chemical-mediated (*M*′), the performance tended to decrease in the case of direct interaction (*D*′). The heatmap on the right side of each panel aids in the comparison between different conditions/methods. Both in Figure 2a,b, the value of row D′5 and column M′5 is positive, which means that the median of LIMITS in *D*′ was higher than that of *M*′ (black dot indicates that the difference is significant (p<0.05) in terms of Mann–Whitney test). On the other hand, CCM was robust to the effect of direct or chemical-mediated interactions compared to LIMITS. In Figure 2a,b, the value of row D’4 and column M’4 shows that the difference between the median of CCM was close to zero and not significant. Relative to LIMITS and CCM, the correlation-based methods, Pearson and Spearman rank correlation and LSA, were ineffective in detecting both types of interactions, with median values always around 0.5. On the other hand, in the absence of nutrient competition (*M* and *D*), it was difficult to detect interactions with any of the methods. None of the methods significantly outperformed the others.

For *M*′ and *D*′, the superiority of LIMITS over other methods was also evident when evaluated using precision (Figure 2c,d). In contrast to the case of ROC-AUC, there was no significant decrease in the performance of LIMITS in ‘*M*’ relative to *D*′. This can be considered superficial for two reasons. The first reason is that precision is a criterion for identified interactions, but validity is not considered for non-identified interactions. The second reason is that it is necessary to set a single threshold for a classifier to obtain a precision value. Since the setting of the threshold is arbitrary, the same result may not always be obtained. Therefore, it was suggested that the effects of mediators could be overlooked in the evaluation by precision. Precision was higher in *M* relative to *D* and *M*′ (Figure 2c,d), but this was due to the difference in connectance since connectance was highest in *M* in all simulation conditions compared to other models (Appendix B; Figure A1) and precision was highly correlated to connectance (Supporting Figure S6). Thus, it is inappropriate to conclude that either the presence of mediators or nutrient competition improved the performance of network inference.

Within the range of the sampling interval (τ) and the magnitude of noise (σ) we studied, the above results were mostly robust, and there were no systematic dependencies of performance of the methods on either of the parameters (see Supporting Figures S7–S13).

## 4. Discussion

We compared the performance of five network inference methods in detecting interactions based on time series when interactions are mediated by chemicals or occur through direct interactions between species, as well as in different competitive contexts. Our results suggest that: (1) the existence of mediators can make microbial interactions difficult to detect, but the degree of difficulty may be method dependent, (2) nutrient competition can play an essential role for the detectability of interactions, and (3) correlation-based methods are not useful for detecting interactions from time series.

Among the methods evaluated in this study, LIMITS, which is derived from the discrete version of the generalized Lotka–Volterra equation, was found to be the most reliable. However, its performance will be reduced if chemical-mediated interactions are dominant due to a mismatch between the processes that the method assumes and those that actually occur. Although the performance of CCM was not as good as LIMITS, CCM would be more robust to the presence of chemical-mediated interactions compared to LIMITS. This may be due to the fact that CCM is not dependent on any specific equation and is based on a nonlinear forecasting method that can flexibly capture the relationship between variables. In summary, LIMITS is recommended as the network inference method in general since it outperformed the other methods under any application conditions in *M*′ and *D*′ and no method clearly outperformed the others in M and D. However, further investigation around the tools of nonlinear forecasting, especially integration with the machine learning framework used in LIMITS to improve its applicability to nonlinear dynamics, will be a promising direction for time-series-based network inference targeting microbial communities, where chemical-mediated interactions are thought to play a major role.

In the models with resource competition (*M*′, *D*′), an increase in one species negatively affects the growth rate of other species and can reduce the population size. Thus, nutrient competition can facilitate coordinated variation in abundance and likely promote the detection of interactions. In fact, the difference between *M*, *D* and *M*′, *D*′ was mostly characterized by the coefficient of variation (Appendix B). In microbiota, there are a few essential nutrients that are common to many species [28], as well as diverse chemicals that mediate interactions. Space will also be an important resource if, for example, the substrate for colonization can be a limiting factor. Thus, while we need to be careful about its strength, the network inference would usually benefit from competitive processes.

Finally, although correlation-based methods are widely used to infer interaction networks from time series, it should be noted that their reliability can be very low, regardless of whether the interactions are direct or chemical-mediated. This has been pointed out repeatedly in previous studies [12,26,29] for direct interactions, but we feel it is important to highlight again.

There are multiple levels of understanding of networks, from properties of the network as a whole, such as degree distribution and average degree, to properties of individual nodes, such as network centrality. The more attention we pay to finer scale properties, the more accurate the network inference needs to be. Therefore, improving the accuracy of the method used for network inference is essential for a network-based understanding of biological systems.

## 5. Conclusions

We found that the existence of mediators can make microbial interactions difficult to detect. However, the degree of difficulty was different among the methods. CCM and LIMITS were capable of detecting interactions from the time series. While LIMITS performed better than CCM, it was less robust to the presence of chemical-mediated interactions. Our result also suggests that the presence of nutrient competition can facilitate the detection of interactions. The existence of chemical-mediated interactions among microbial species poses a new challenge to overcome for the development of a network-based understanding of microbiomes and their relationship to hosts and the environment. Our study would provide an in silico experimental system of microbial population dynamics, including chemical-mediated interactions, to evaluate network inference methods that will be developed in the future.

## Figures and Tables

**Table 1:** Model parameters. Here, $u_{p}\left(x,y\right)$ means that the numbers are randomly drawn from a uniform distribution between x and y with probability *p*, and otherwise zero.

	Description	Value
*n*	Number of microbes	10
*m*	Number of chemicals	5
K	Carrying capacity	1
δ	Dilution rate	0.01
$r_{i}$	Growth rate	$u_{1}\left(0.05,\;0.5\right)$
$\kappa_{ik}$	Half-saturation density	$u_{1}\left(0.5,\;1.5\right)\times 10^{-3}$
$\rho_{ik}{}^{+}$	Positive effect of chemicals on microbes	$u_{0.2}\left(0.05,\;0.5\right)$
$\rho_{ik}{}^{-}$	Negative effect of chemicals on microbes	$u_{0.2}\left(0.05,\;0.5\right)$
$\alpha_{ki}$	Maximum consumption rate of chemicals	$u_{0.2}\left(0.5,\;1.5\right)$
$\beta_{ki}$	Production rate of chemicals	$u_{0.2}\left(0.05,\;0.15\right)$
ϵ	Influx of microbes	$10^{-7}$

**Table 2:** Simulation parameters.

Name	Description	Value
N	Number of time series in a data set	288
$M_{p}$	Number of pairs in each generation	32
$m_{p}$	Number of parents for next generation	4
$t_{max}$	Length of time series generated by simulation	10,000
$t_{0}$	Length of time series discarded as the initial transient	2000
$T_{max}$	Number of iterations of the optimization procedure	60 (for *M* and *D*) 120 (for *M*′ and *D*′)
η	Criterion for major species	$10^{-2}$
ω	Threshold value for the evaluation function	5

## Data Availability

The data and computer codes for this study are available as supplementary information.

## Supplementary Materials

The following supporting information can be downloaded at: https://www.mdpi.com/article/10.3390/ijerph19031228/s1, Figure S1. Evaluation criteria for binary classification problems and their relationship to ROC curves; Figure S2. Representative result of network inference with the 5 methods for model M (τ=40 and σ=1); Figure S3. Representative result of network inference with the 5 methods for model D (τ=40 and σ=1); Figure S4. Representative result of network inference with the 5 methods for model M’ (τ=40 and σ=1); Figure S5. Representative result of network inference with the 5 methods for model D’ (τ=40 and σ=1); Figure S6. Relationship between the basic properties of a community and the performance of network inference for different pairs of model and method; Figure S7. Relationship between the parameters for generating data sets (sampling interval τ and magnitude of noise σ ) and the performance of network inference for different pairs of model and method; Figure S8. Performance of network inference methods for different models (left) and the comparison of the different pairs of model and method (right) for τ=10 and σ=1; Figure S9. Performance of network inference methods for different models (left) and the comparison of the different pairs of model and method (right) for τ=20 and σ=1; Figure S10. Performance of network inference methods for different models (left) and the comparison of the different pairs of model and method (right) for τ=80 and σ=1; Figure S11. Performance of network inference methods for different models (left) and the comparison of the different pairs of model and method (right) for τ=40 and σ=0.5; Figure S12. Performance of network inference methods for different models (left) and the comparison of the different pairs of model and method (right) for τ=40 and σ=2; Figure S13. Performance of network inference methods for different models (left) and the comparison of the different pairs of model and method (right) for τ=40 and σ=4.

## Appendix A. Implementation of CCM for Network Inference

To perform CCM, it is necessary to determine the appropriate embedding dimension for the state space reconstruction. We applied the pseudo-neighborhood method (Cao 1997) to each variable to determine the embedding dimension. Then, the strength of the causal relationship between *X* and *Y* was obtained as follows. First, we prepared the state space $M_{Y}$ reconstructed by the Y’s embedding dimension $\varepsilon_{Y}$. Then, we predict X from $M_{Y}$ using the simplex projection method (Sugihara et al. 2012), which is labeled as $X^{Y}$. The cross-map prediction skill $\rho_{X\to Y}$ is the Pearson’s correlation coefficient between X and $X^{Y}$. To calculate the *p*-value, we prepared ${\bar{M}}_{Y}$ where half of the points in $M_{Y}$ were sampled without replacement and repeated the same procedure as above to obtain the predictive skill. We repeated this procedure N=2000 times to obtain ${\bar{\rho }}_{X\to Y}=\left\{{\bar{\rho }}_{X\to Y}{}^{\left(i\right)}\right\}$, where ${\bar{\rho }}_{X\to Y}{}^{\left(i\right)}$ is the predictive skill obtained in the ith iteration. The *p*-value of $\rho_{X\to Y}$ is obtained as the number of ${\bar{\rho }}_{X\to Y}{}^{\left(i\right)}$ satisfying $\rho_{X\to Y}^{\left(i\right)}\geq \rho_{X\to Y}$ divided by N. To determine the presence of a causal relationship, ρ must be greater than zero and increases with the number of data points in the reconstructed state space. Here, we evaluated whether ρ is increasing by limiting the number of data points when obtaining ${\bar{\rho }}_{X\to Y}$ and compared it to $\rho_{X\to Y}$ [30].

## Appendix B. Basic Characteristics of Communities

We generated 288 virtual microbial communities with 7 different parameter sets ((τ,σ)∈{(10,1), (20,1),(40,1),(80,1),(40,0.5),(40,2),(40,4)}) for each model. The characteristics of the communities were adjusted based on the evaluation function (Equation (8)) but differed according to the factors inherent in each model (Figure A1). In *M*, it is likely that both the number of major species and Sympson’s *D* were easy to adjust because the abundance of a species does not directly affect the abundance of other species. In other cases, at least either the direct species interactions (together with the requirement on the connectivity between major species) or nutrient competition can limit coexistence. Particularly for direct species-species interaction, the higher the connectance, the more difficult it is for a system to achieve multispecies coexistence (May 1973). Therefore, it is natural for *D* and *D*′ to have a connectance smaller than 0.5. On the other hand, in *M* and *M*′, one species can consume a chemical and reduce its impact on another species. This lowers the strength of the interaction, and thus stable coexistence can be maintained even at relatively high connectance. The coefficient of variation was higher in *M*′ and *D*′ than in M and *D*. In *M*′ and *D*′, an increase in one species negatively affects the growth rate of the other species and can reduce the population size; this facilitates the oscillation of abundance both as an intrinsic dynamic of the system and as a noise response [31].
