# Supplementary material for: Chemical-Mediated Microbial Interactions Can Reduce the Effectiveness of Time-Series-Based Inference of Ecological Interaction Networks
Source: Int J Environ Res Public Health. 2022 Jan 22;19(3):1228. doi: 10.3390/ijerph19031228 (PMC8834966; doi:10.3390/ijerph19031228)
Supplement: Supplementary file 1 [file ijerph-19-01228-s001.zip › ijerph-1530784-supplementary.pdf]

Supplementary materials - Supporting figures

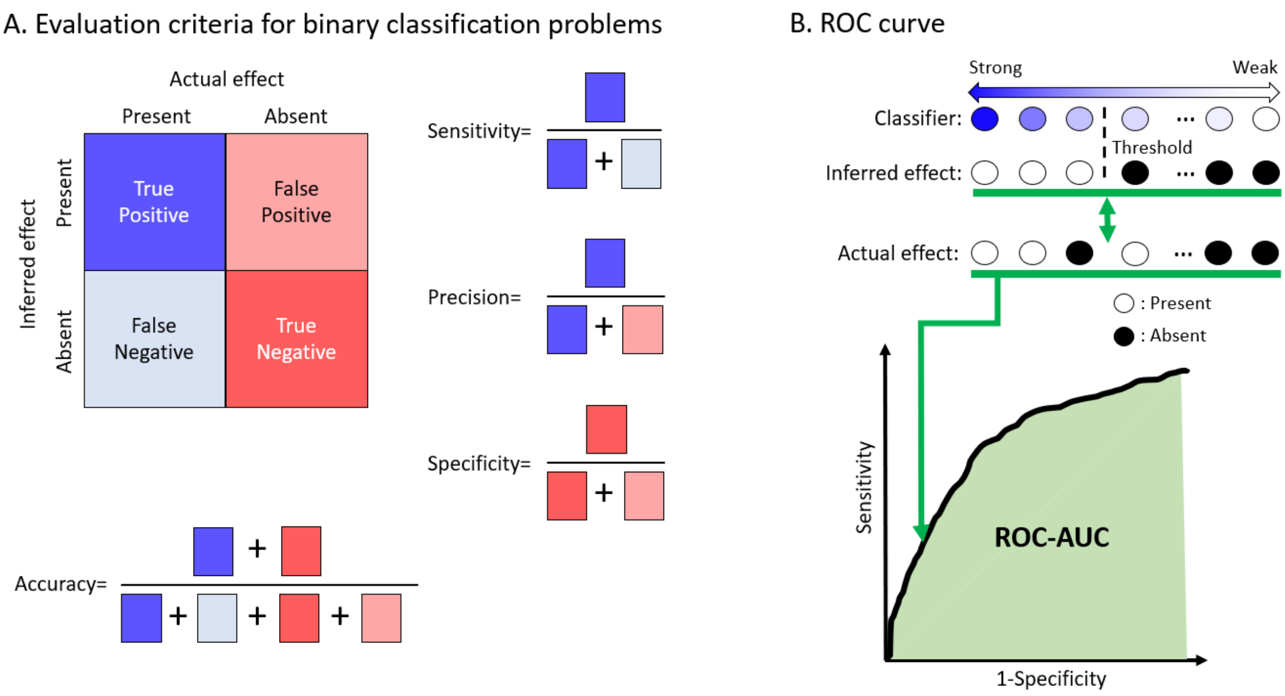

Figure S1. Evaluation criteria for binary classification problems and their relationship to ROC curves. A. evaluation criteria for binary classification problems. Here, an effect from a species on another is represented by a non-zero value of the off-diagonal elements of the actual/inferred interaction matrix. True positive (TP) represents a result of inference that correctly indicates the presence of an effect from one species to another, true negative (TN) represents a result of inference that correctly indicates the absence of an effect from one species to another, false positive (FP) represents a result of inference which incorrectly indicates that an effect is present from one species to another, and false negative (FN) is a result of inference which incorrectly indicates that an effect from one species to another is absent. B. Explanation of ROC curve. Here, a circle corresponds to an off-diagonal element of an interaction matrix. Suppose we set a threshold value for a classifier and determine that effects from one species to another corresponding to elements above the threshold value are present, and elements below the threshold value are absent. By matching this with the corresponding elements of the actual interaction matrix, we can calculate sensitivity and specificity and plot a single point on the 2D plane. The ROC curve is obtained by repeating the calculation with changing threshold values, and the ROC-AUC is the area underneath the curve.

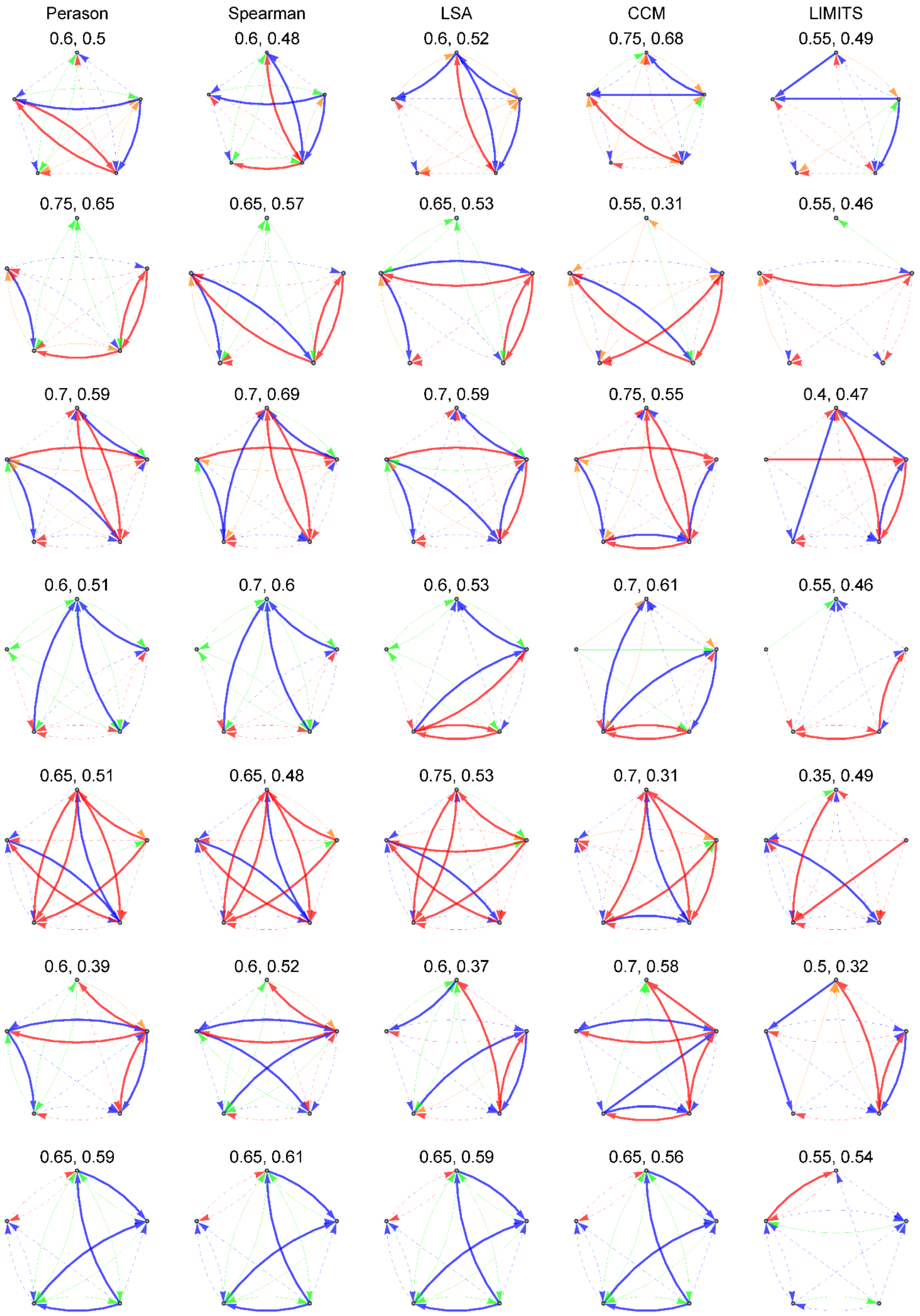

Figure S2. Representative result of network inference with the five methods for model M ($\tau = 40$ and $\sigma = 1$). Blue and red arrows indicate the positive and negative interaction between species in the actual (effective) interaction matrix, respectively, and green and orange arrows indicate the positive and negative interaction between species in the estimated interaction matrix. Thick arrows indicate correctly detected interactions. The values above the graph show the precision and accuracy in this order.

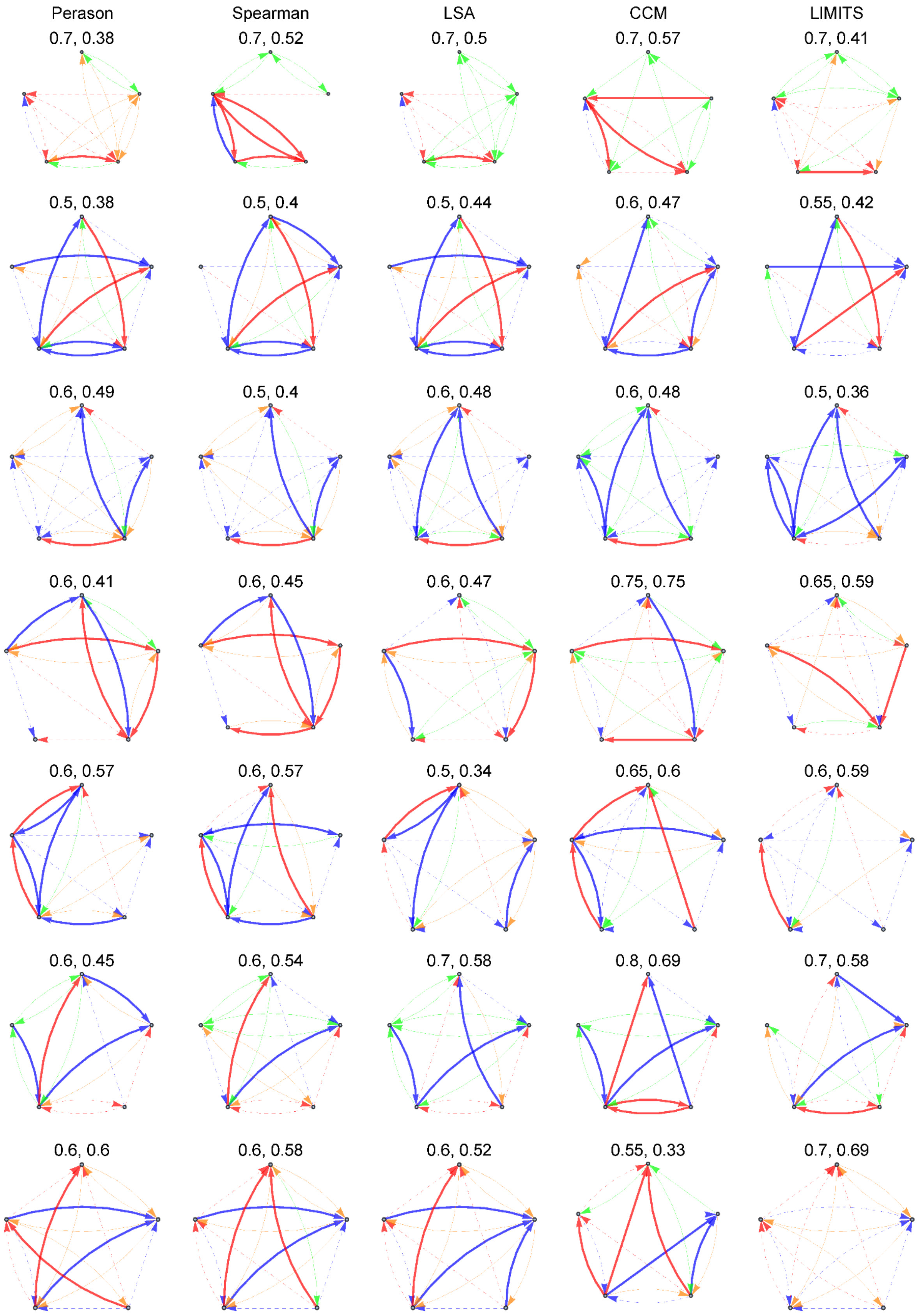

Figure S3. Representative result of network inference with the five methods for model D (τ = 40 and σ = 1). See legend of figureS1 for details.

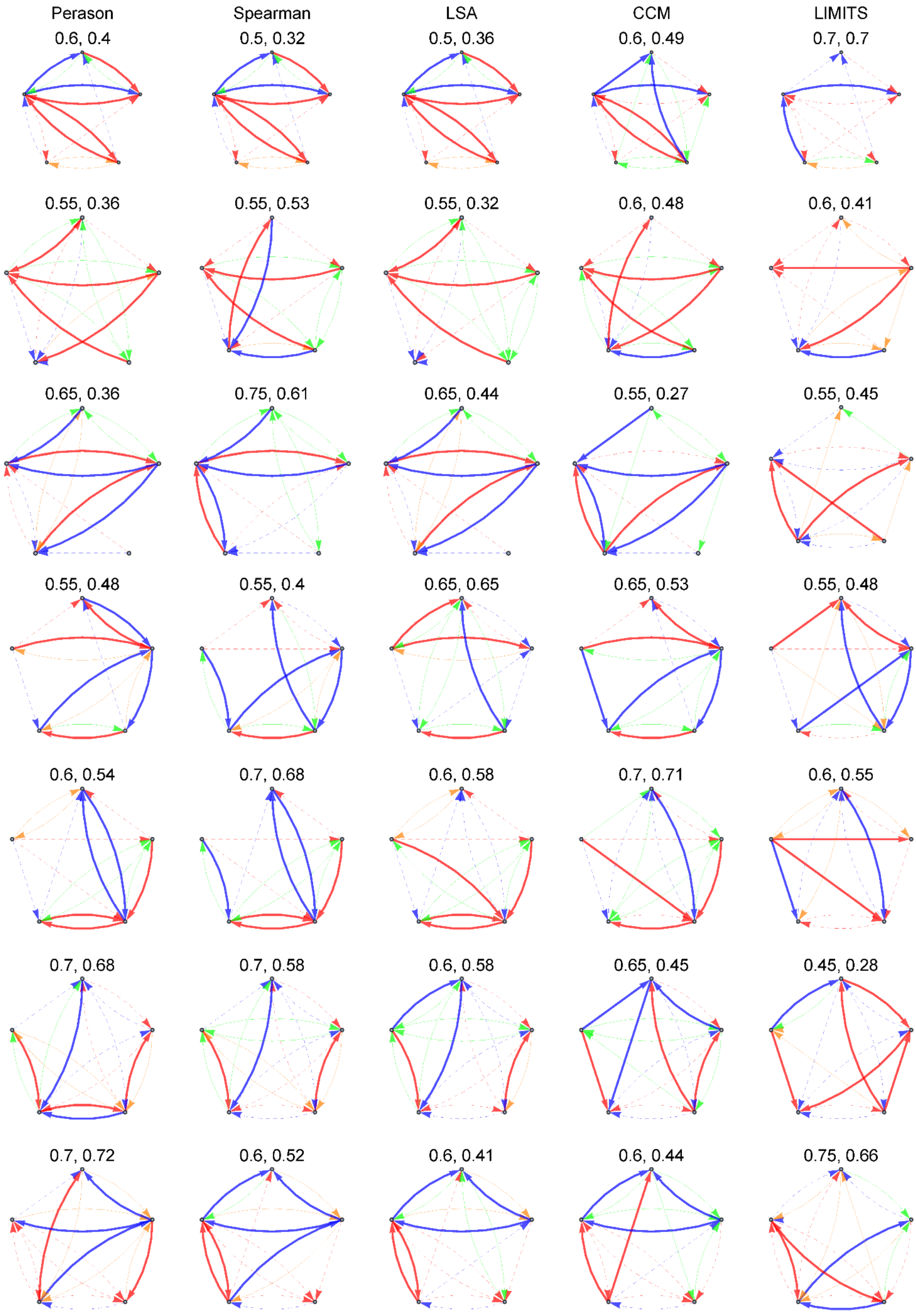

Figure S4. Representative result of network inference with the five methods for model M′ (τ = 40 and σ = 1). See legend of figureS1 for details.

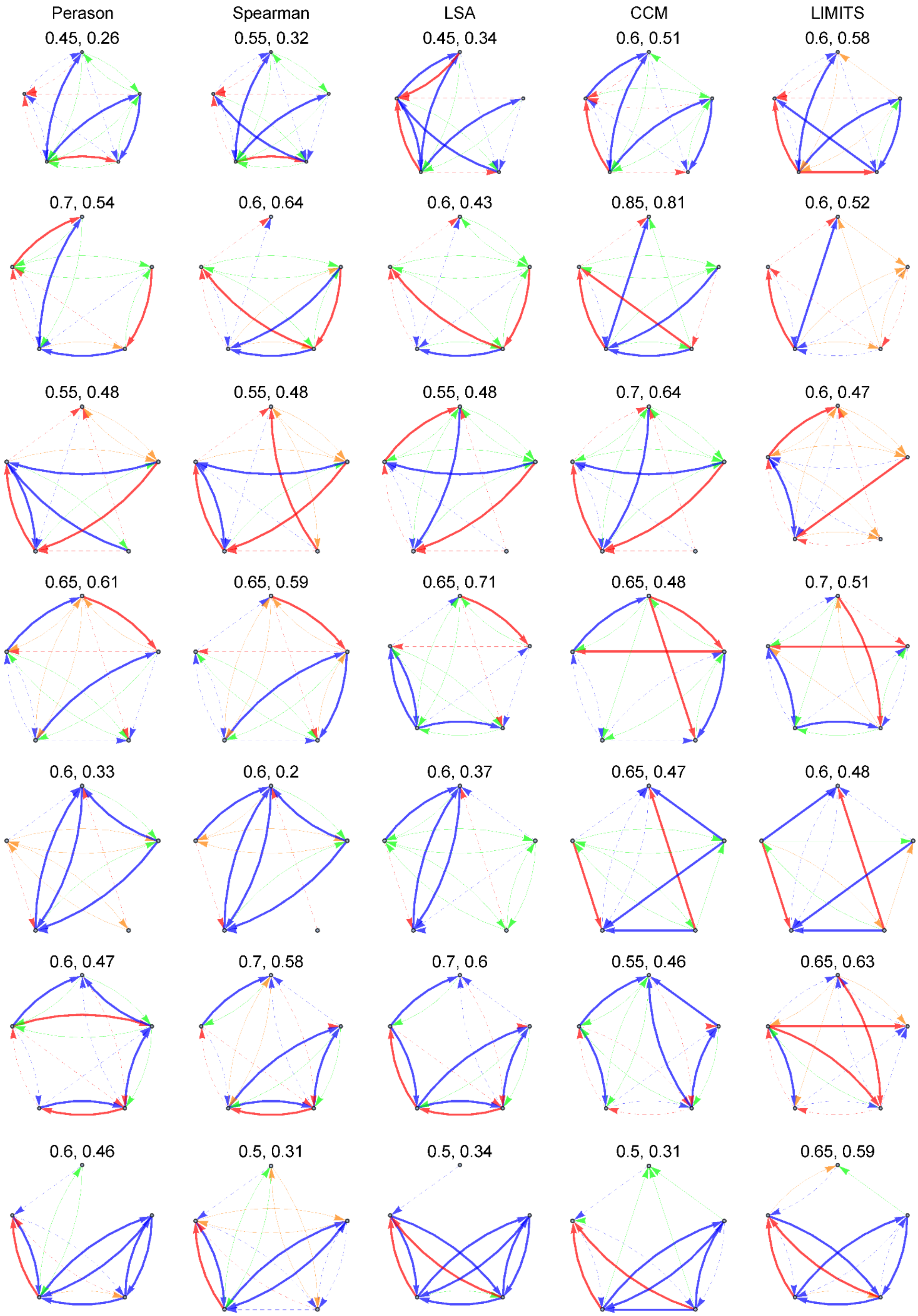

Figure S5. Representative result of network inference with the five methods for model D′ ($\tau = 40$ and $\sigma = 1$). See legend of figureS1 for details.

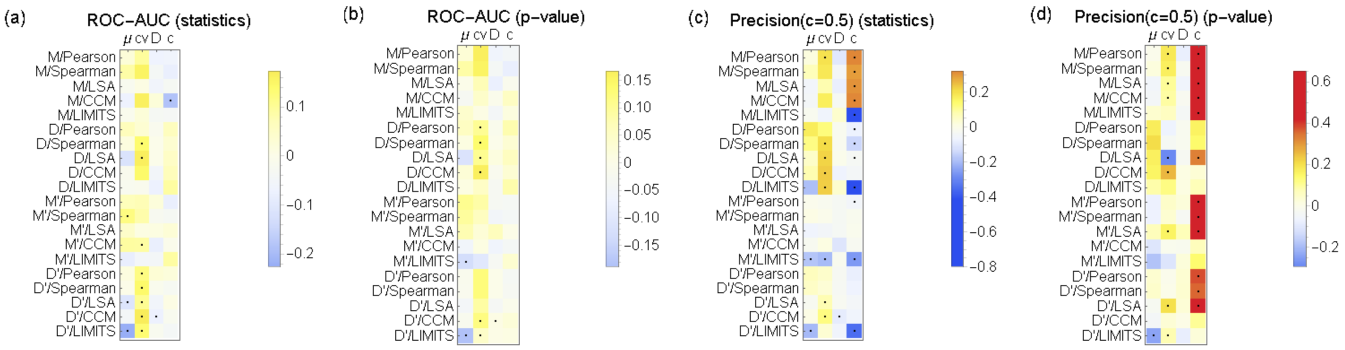

Figure S6. Relationship between the basic properties of a community and the performance of network inference for different pairs of model and method. Correlations between the basic properties and the performance measured by each evaluation criterion for 288 communities in the reference condition ($\tau = 40$ and $\sigma = 1$) are shown. Black dots indicate significant ($p < 0.05$) correlation. μ is the mean abundance $\hat{x}_i$ averaged over the top five abundant species, cv, D, c is the coefficient of variation, the Simpson's diversity index, and connectance, respectively, and calculated for the top five abundant species.

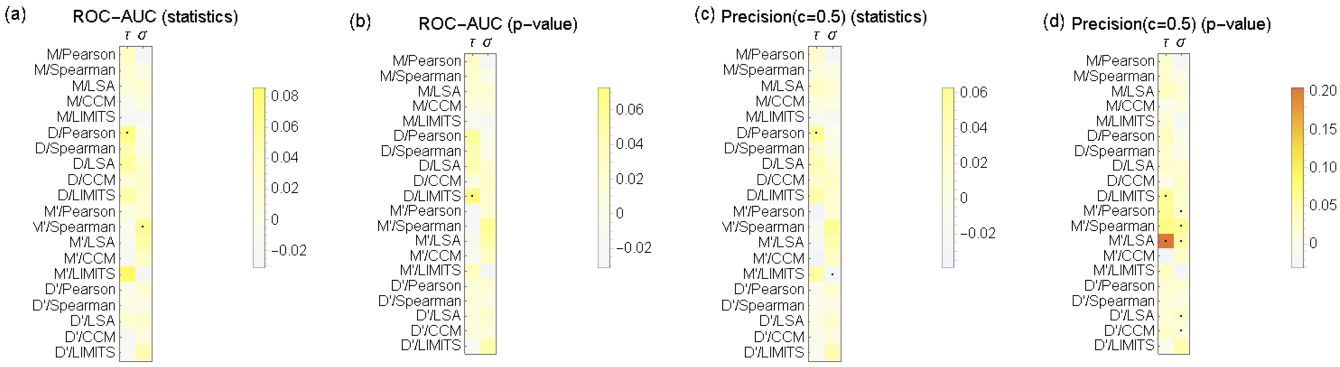

Figure S7. Relationship between the parameters for generating data sets (sampling interval τ and magnitude of noise σ) and the performance of network inference for different pairs of model and method. In both τ and σ, correlations were calculated for four different conditions ($\tau \in \{10,20,40,80\}$ with a fixed noise magnitude $\sigma = 1$, and $\sigma \in \{0.5,1,2,4\}$ with a fixed sampling interval $\tau = 1$) where each of conditions contains 288 data points. Black dots indicate significant ($p < 0.05$) correlation.

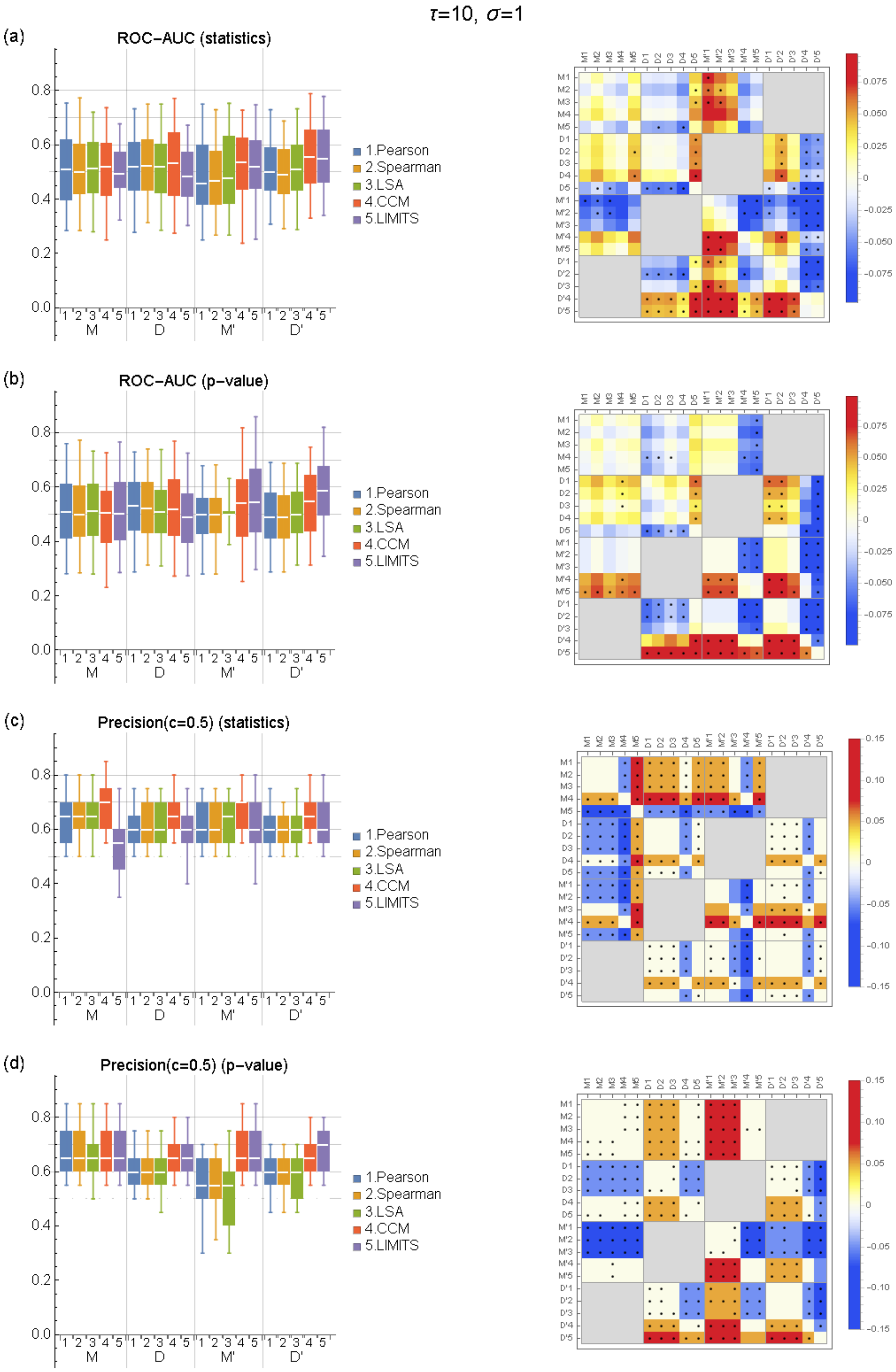

Figure S8. Performance of network inference methods for different models (left) and the comparison of the different pairs of model and method (right) for $\tau = 10$ and $\sigma = 1$. See legend of figure1 for details.

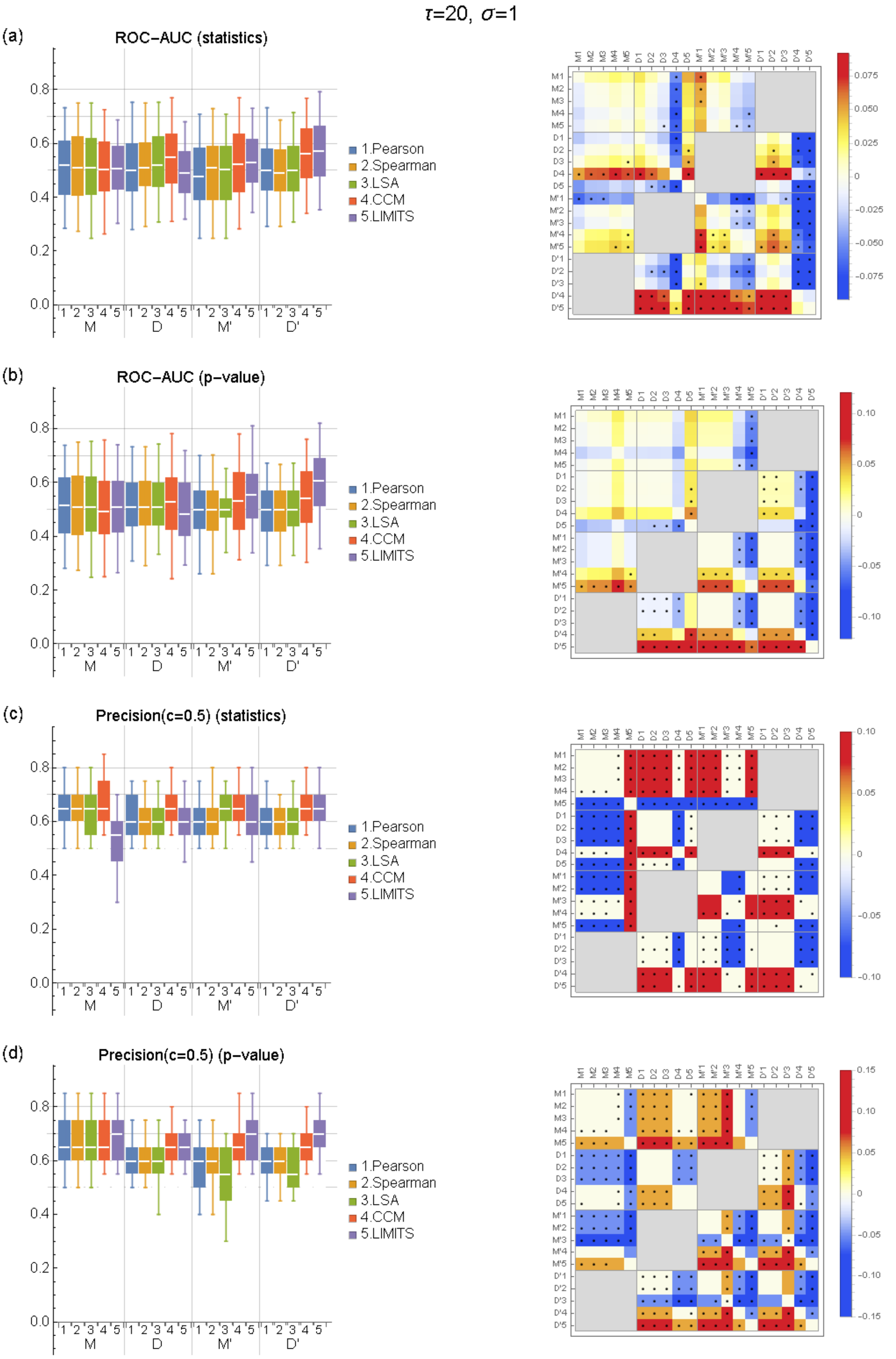

Figure S9. Performance of network inference methods for different models (left) and the comparison of the different pairs of model and method (right) for $\tau = 20$ and $\sigma = 1$. See legend of figure1 for details.

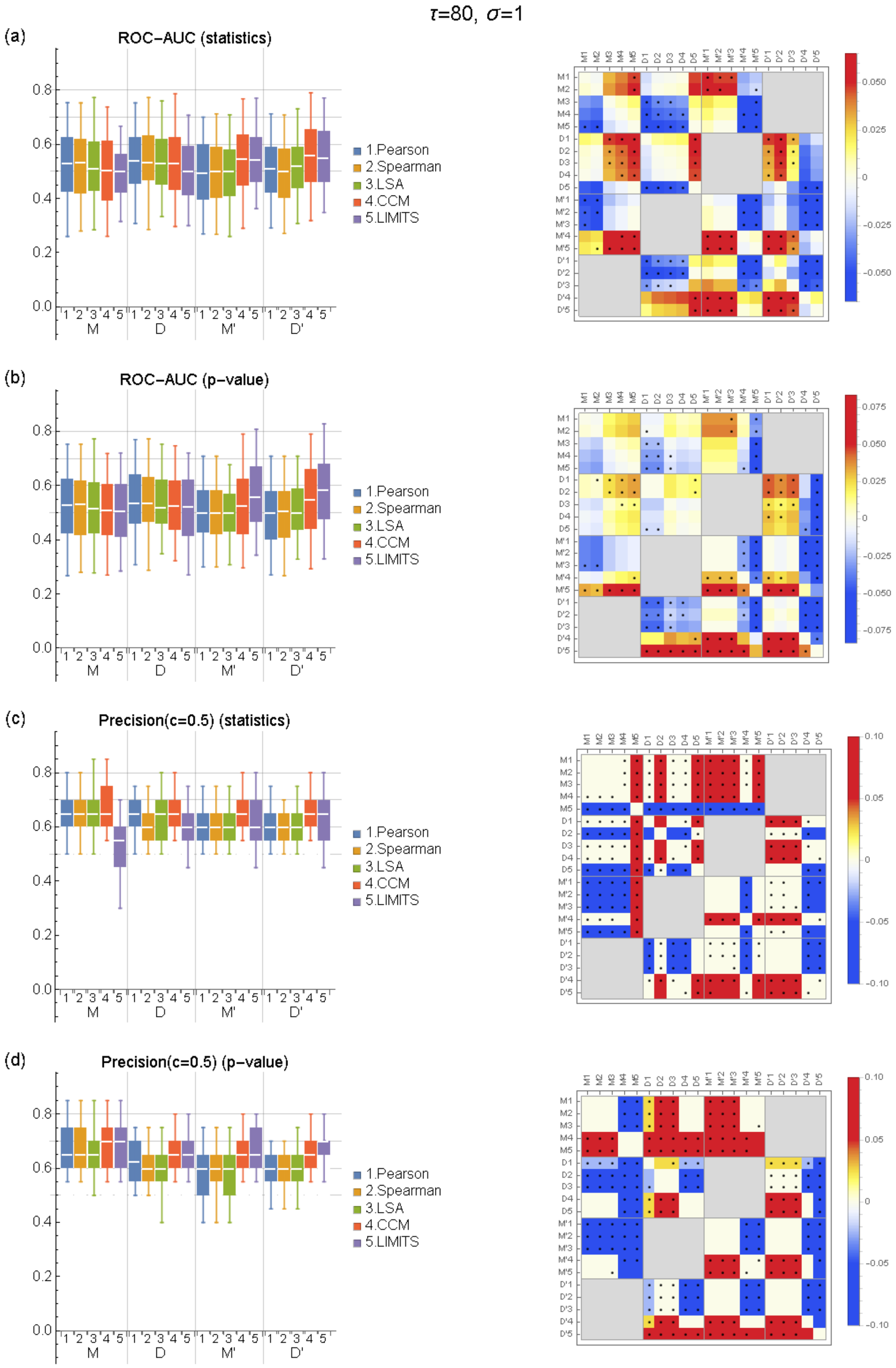

Figure S10. Performance of network inference methods for different models (left) and the comparison of the different pairs of model and method (right) for $\tau = 80$ and $\sigma = 1$. See legend of figure1 for details.

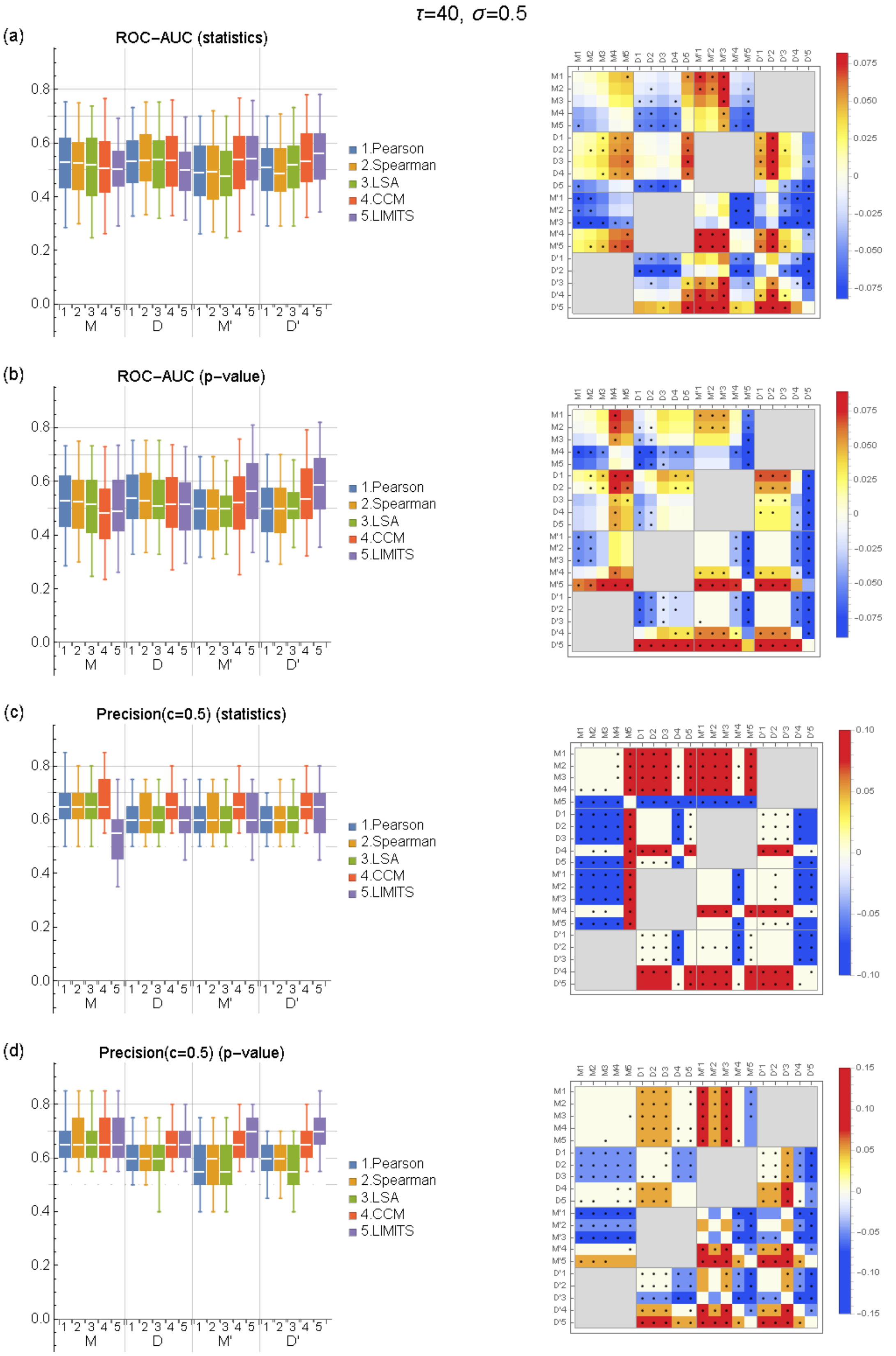

Figure S11. Performance of network inference methods for different models (left) and the comparison of the different pairs of model and method (right) for $\tau = 40$ and $\sigma = 0.5$. See legend of figure1 for details.

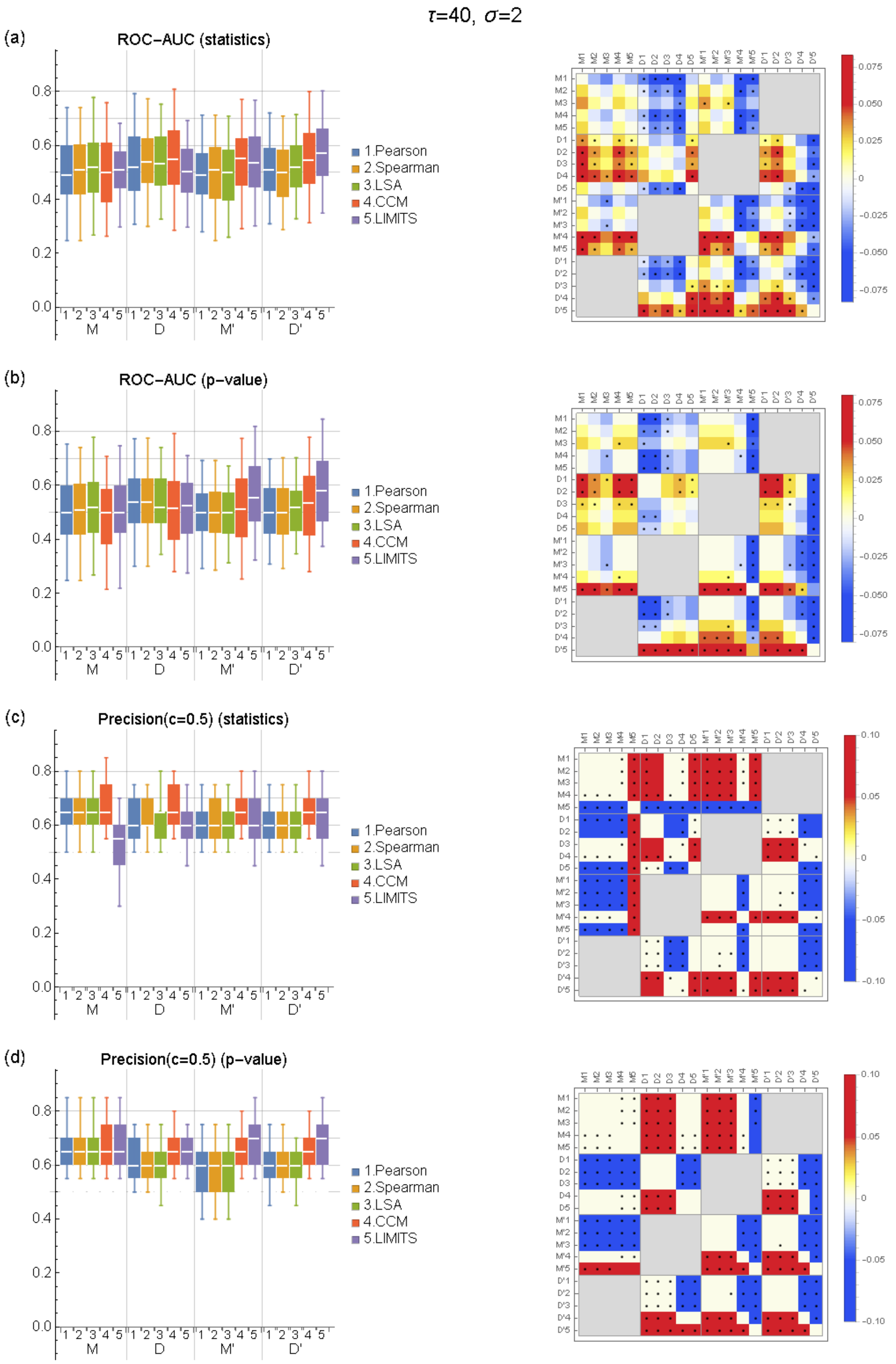

Figure S12. Performance of network inference methods for different models (left) and the comparison of the different pairs of model and method (right) for $\tau = 40$ and $\sigma = 2$. See legend of figure1 for details.

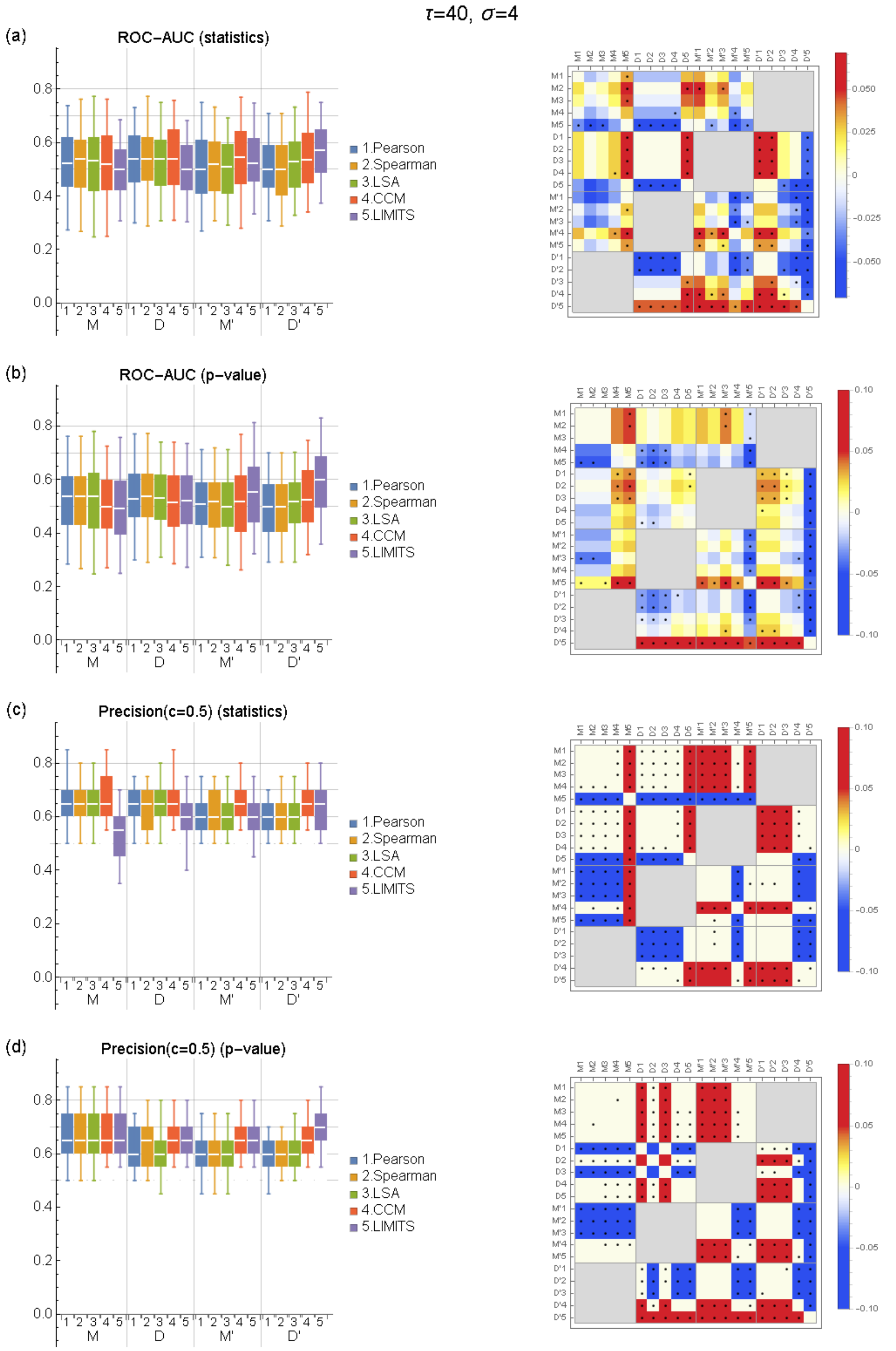

Figure S13. Performance of network inference methods for different models (left) and the comparison of the different pairs of model and method (right) for $\tau = 40$ and $\sigma = 4$. See legend of figure1 for details.
